# Predictors of recurrent wheezing in late preterm infants

**DOI:** 10.1002/ppul.26739

**Published:** 2023-11-03

**Authors:** Brooke Gustafson, Rodney D. Britt, Mariah Eisner, Deepika Narayanan, Mitchell H. Grayson

**Affiliations:** 1 Division of Pulmonary Medicine, Nationwide Children’s Hospital, Columbus, Ohio, USA; 2 Department of Pediatrics, Center for Perinatal Research, The Abigail Wexner Research Institute at Nationwide Children’s Hospital, The Ohio State University, Columbus, Ohio, USA; 3 Department of Pediatrics, The Ohio State University College of Medicine, Columbus, Ohio, USA; 4 Biostatistics Resource at Nationwide Children’s Hospital, Columbus, Ohio, USA; 5 Department of Pediatrics, Division of Allergy and Immunology, Nationwide Children’s Hospital, Columbus, Ohio, USA; 6 Center for Clinical and Translational Research, The Abigail Wexner Research Institute at Nationwide Children’s Hospital, Columbus, Ohio, USA

**Keywords:** prematurity, recurrent wheezing

## Abstract

**Introduction:**

Premature infants have an increased risk of respiratory morbidity, including the development of recurrent wheezing. We sought to determine perinatal factors in late preterm infants associated with an increased risk of recurrent wheezing in the first 3 years of life.

**Methods:**

A retrospective chart review of infants born between 32 and 36 weeks gestational age at a tertiary hospital from 2013 to 2016 was performed. Infants with any co-morbid medical conditions were excluded. Recurrent wheezing was identified by two or more visit diagnoses for reactive airway disease, wheezing-associated respiratory infection, wheezing, or asthma during the first 3 years of life. Those with recurrent wheezing were compared to matched preterm infants who did not develop wheezing.

**Results:**

Three hundred and fourteen late preterm infants were included in this study; 210 infants developed recurrent wheezing while 104 did not. Gender, sex, and race were comparable between both groups. Development of wheezing was associated with positive family history of asthma (*p* = .014), receiving antibiotics during the neonatal period (*p* < .001), requiring continuous positive airway pressure for <24 h (*p* = .019), and receiving supplemental oxygen during the newborn period (*p* = .023).

**Conclusion:**

This study retrospectively identified risk factors associated with development of wheezing in late preterm infants. Prospective studies are needed to determine whether these factors will predict recurrent wheeze in this patient population.

## 1 | INTRODUCTION

Incidence of preterm birth, defined as birth before 37 weeks gestational age, has significantly increased worldwide over the last few decades.^1^ Preterm births now account for approximately 10% of live births in the United States annually, and there are over 15 million preterm births worldwide every year.^2^ With advancements in prenatal and neonatal medical care, survival rates in this patient population have improved significantly.^3^ Therefore, understanding long-term health outcomes in this group has become increasingly important. Chief among these outcomes is respiratory morbidity and mortality associated with preterm birth.

Preterm infants have an increased risk for the development of several respiratory disorders, including bronchopulmonary dysplasia (BPD), chronic respiratory failure, and wheezing-related disorders including asthma.^3^ Extremely premature infants are at highest risk for significant respiratory morbidity. In this group specifically, BPD is the most common chronic lung disease and is associated with increased mortality, respiratory morbidity, adverse neurodevelopmental outcomes, and increased healthcare costs.^4^ Late preterm infants, those born between 32 and 36 weeks gestational age, are less likely to develop BPD; however, these infants are still at an increased risk of adverse respiratory outcomes.^5^ Chief among these is the development of bronchial hyperactivity presenting as isolated or recurrent wheezing and later development of asthma.^5,6^ A previous systematic review found an increased risk of wheezing disorders in preterm babies, with an inverse correlation between gestational age at birth and the risk of wheezing.^6^ Another study found that more than 54% of infants born at 32–36 weeks gestation had a physician diagnosis of wheezing within the first 3 years of life, with nearly half of these infants having recurrent wheezing.^7^ Compared to term infants, this wheezing results in increased healthcare utilization and respiratory morbidity.^8^

Several studies of preterm infants have identified potential risk factors that may lead to the development of recurrent wheezing after the neonatal period.^5,9^ One study found that lower gestational age, family history of asthma, and maternal tobacco use were associated with the development of bronchial hyperactivity and asthma.^3,5^ Another observational cohort of children with postprematurity respiratory disease studying perinatal factors in more detail found those exposed to mechanical ventilation and postnatal steroids had evidence of worsening obstruction in lung function testing throughout childhood.^10^ Racial and ethnic disparities related to preterm birth and recurrent wheezing are also well described, with non-Hispanic black race being a consistent risk factor for preterm birth and adverse pregnancy outcomes in the United States compared with non-Hispanic White race.^11^ Additionally, it is known that African American/black and Hispanic children are at increased risk for asthma compared with white children, with onset occurring earlier in childhood.^12^

Ultimately though, differences in definitions of wheezing outcomes, study design, and study populations with a large range of gestational ages have made comparisons between studies difficult. Despite this, it is clear the development of bronchial hyperactivity is a highly complex, multifactorial process. Understanding risk factors and mechanisms leading to the development of recurrent wheezing is essential to find targets for both primary prevention and future interventions and treatments.

The purpose of this study was to assess risk factors specifically in late preterm infants that are associated with the development of recurrent wheezing during the first 3 years of life. We conducted a retrospective review of late preterm infants born over a 3-year period at our tertiary care center who developed recurrent wheezing. This group was compared to preterm infants born over the same time frame who did not develop recurrent wheezing. Factors related specifically to the newborn period, including medication exposure and the need for respiratory support, were reviewed and compared between groups. Previously identified risk factors, such as family history of asthma and tobacco exposure, were also compared between groups. Our primary aim was assessing differences in exposures between groups. In infants who developed recurrent wheezing, the presence of a viral source at first-ever wheezing episode was also recorded.

## 2 | METHODS

### 2.1 | Study design and patient population

This retrospective chart review included late preterm infants between 32 and 36 weeks gestational age who received treatment at Nationwide Children’s Hospital from 2013 to 2016 who developed recurrent wheezing during the first 3 years of life. This cohort was ascertained through an electronic medical record review of all infants admitted to Nationwide Children’s Hospital’s neonatal intensive care units (NICUs) over the designated 3-year period with a gestational age of 32–36 weeks. A 3-year follow-up period was selected to assess risk factors before school entry. Any infants with co-morbid conditions including BPD, gastroesophageal reflux disease, aspiration, tracheobronchomalacia, tracheostomy dependence, hypoxic-ischemic encephalopathy, epilepsy, congenital heart disease, genetic diseases, or identified congenital anomalies were excluded from the study. These comorbidities were identified by the presence of international classification of diseases (ICD-10 th revision) codes for the diagnoses within the electronic medical record. Late preterm infants without any comorbid diagnoses were separated into two groups: those who developed recurrent wheezing and those who did not. Recurrent wheezing was defined as having two or more healthcare visits using ICD-10 codes for wheezing, reactive airway disease, wheezing-associated respiratory infection, or asthma at any point during the first 3 years of life. The healthcare visits included emergency department, urgent care, subspecialty clinic, and primary care provider visits, but the specific visit type was not captured for this review. This recurrent wheezing group was compared to the remaining group of late preterm infants born during the same time frame who did not meet the criteria for the development of recurrent wheezing. A patient database was created to collect demographic and clinical information via electronic medical record chart review for both groups.

Demographic information including gender, sex, and race were included for all patients. Birth weight, method of delivery (vaginal delivery vs. Cesarean section), history of maternal tobacco use during pregnancy, and family history of asthma were included for all infants. Perinatal exposures, those from birth to 7 days of age, were also included. Perinatal medication exposures (antibiotics, surfactant, steroids, albuterol), perinatal respiratory support (invasive mechanical ventilation, noninvasive mechanical ventilation, supplemental oxygen), perinatal bacterial and viral testing results, and pertinent environmental exposures after birth (tobacco) were recorded for all infants when available. Duration of noninvasive mechanical ventilation in the form of continuous positive airway pressure (CPAP) and supplemental oxygen were categorized, and categories were analyzed only for subjects who received these supports in the newborn period. For infants who developed recurrent wheezing, viral organism at initial presentation was recorded if any type of viral testing was performed and a positive source was identified.

### 2.2 | Statistical analysis

Variables were visualized using bar and violin plots to check data quality and evaluate distributional assumptions. Data were summarized using frequency (percentage) for categorical variables, mean (standard deviation [SD]) for continuous symmetric variables, and median (interquartile range [IQR]) for continuous skewed variables. Group comparisons between patients with and without wheezing disorders were conducted with the χ^2^ or Fisher’s exact test for categorical variables, two-sample *t* test for normally distributed continuous variables, and Wilcoxon rank sum test for skewed continuous variables. Fisher’s exact test was used for categorical variables with expected cell counts <5 and χ^2^ for ≥5. Unadjusted odds ratios (ORs) predicting the probability of developing recurrent wheezing were reported for categorical variables. Thirty-two weeks gestational age, female sex, and non-Hispanic White race were used as reference levels. Independent variables with *p* ≤ .2 were evaluated in a multivariable logistic regression model for developing recurrent wheezing. The final model was chosen through backward stepwise selection based on the Akaike information criterion (AIC) to eliminate insignificant predictors. Adjusted ORs were presented with 95% confidence intervals (CIs). Two-sided *p* < .05 were considered statistically significant. All statistical analyses were performed in R version 4.0 (R Core Team) with reproducible programming in R Markdown.

### 2.3 | Ethical statement

This study was approved by the Nationwide Children’s Hospital Institutional Review Board (Protocol ID: STUDY00002049).

## 3 | RESULTS

There were 314 late preterm infants without any comorbid diagnoses born between 2013 and 2016 included in this retrospective study. Among 314 subjects, 210 infants developed recurrent wheezing during the first 3 years of life, and the remaining 104 infants did not. Patient gestational age, sex, race, birth weight, length of NICU admission, and method of delivery are presented in Table 1. Sex, race, and gender between groups were similar. The median NICU length of stay and mean birth weight were also comparable between groups.

Differences in family history, tobacco exposure, medication use during the neonatal period, and need for respiratory support in the neonatal period are reported in Table 2. In subjects with recurrent wheezing, 109 (52%) had a positive family history of asthma, compared to 39 (38%) subjects in the nonwheezing group (*p* = .014). Thirty infants (14%) in the recurrent wheezing group had positive maternal tobacco use during pregnancy, compared to 12 infants (12%) in the nonwheezing group (*p* = .5). Overall, few infants in either group received steroids, surfactant, or albuterol during their neonatal course, and no significant differences were noted between the groups for these treatments. However, 53 infants (25%) in the recurrent wheezing group received antibiotics during the neonatal course, versus eight infants (7.7%) in the nonwheezing group (*p* < .001). When viral or bacterial testing was performed in the neonatal period, organisms were rarely identified (only found in three patients), so these variables were not included in results.

In terms of respiratory support during the newborn period, few infants in either group required invasive mechanical ventilation (34 vs. 10, wheezing vs non-wheezing, respectively; *p* = .11). Seventy-eight infants (37%) in the wheezing group and 29 infants (28%) in the nonwheezing group required CPAP at some point during the neonatal period (*p* = .11). Thirty-four infants (16%) in the wheezing group and seven infants (6.7%) in the nonwheezing group required CPAP support for under 24 h (*p* = .019). Forty-four infants (21%) in the wheezing group and 22 infants (21%) in the nonwheezing group required CPAP support for more than 24 h (*p* > .9). Forty-four infants (21%) needed supplemental oxygen in the recurrent wheezing group, versus 11 infants (11%) in the nonwheezing group (*p* = .023). The duration of supplemental oxygen use (0–48, 48–96, or >96 h) did not correlate with increased odds of developing wheezing. Overall, 98 infants (47%) in the wheezing group required some form of respiratory support (invasive ventilation, CPAP, or supplemental oxygen) during the immediate newborn period, versus 36 infants (35%) in the non-wheezing group (*p* = .042).

**FIGURE 1:**
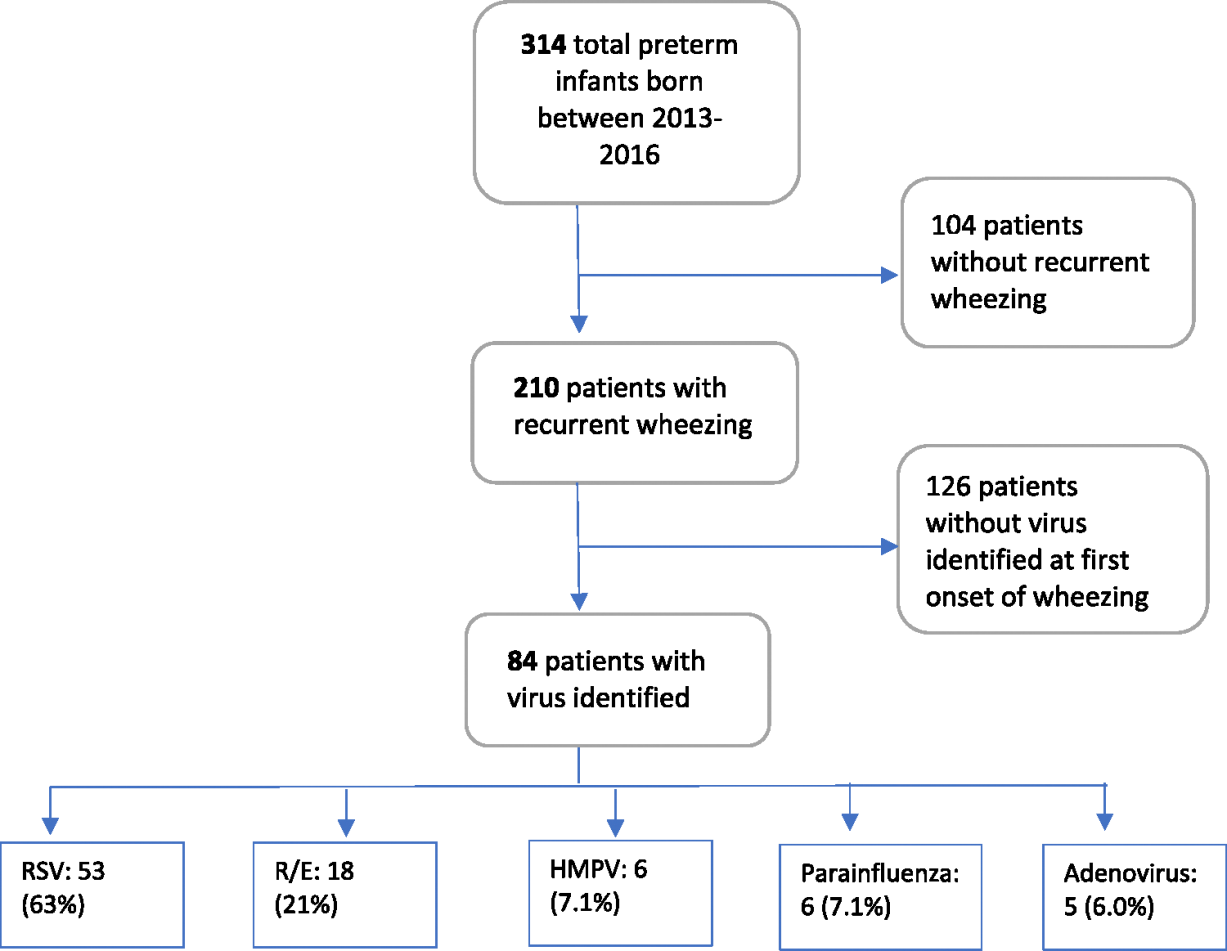
Flow diagram of viral source identification at first presentation in patients with recurrent wheezing. HMPV, human metapneumovirus; R/E, rhino/enterovirus; RSV, respiratory syncytial virus.

In infants who developed recurrent wheezing, 84 of 210 patients (40%) had positive viral testing at their first wheezing presentation (Figure 1). Of those patients, respiratory syncytial virus (RSV) was the most identified virus (53/84, 63%), followed next by rhino/enterovirus (18/84, 21%) and human metapneumovirus (6/84, 7.1%). In our study, tobacco exposure after the newborn period was not nearly associated with increased likelihood of recurrent wheezing (66/210 31%, vs. 22/104, 21%; *p* = .056).

Overall, family history of asthma, antibiotics received during hospitalization after birth, and supplemental oxygen use during the newborn period remained statistically significant after adjusting for NICU length of stay, patient race, CPAP use for <24 h during the neonatal period, and smoking exposure after birth in a multivariable logistic regression model for developing a wheezing disorder (Table 3). History of breastfeeding, having any microbiologic testing obtained during the neonatal period, and need for mechanical ventilation were not selected for inclusion in the final multivariable model based on AIC.

## 4 | DISCUSSION

The aim of this retrospective study was to identify predictive factors associated with the development of recurrent wheezing in late preterm infants based on parental characteristics, perinatal exposures, and postnatal exposures. We did not find that gender, method of delivery, or birth weight conferred an increased risk to the development of wheeze between groups in our study. In review of parental characteristics between groups, however, we found family history of asthma to be a statistically significant predictor for the development of recurrent wheezing in this patient population. Several studies have identified family history of asthma as a strong predictive factor for the development of wheezing and asthma in preterm infants, and this was also evident in our study.^3,5,13,14^ Notably, both patient populations did have high rates of family history of asthma in our study compared to population norms, which may reflect a degree of bias intrinsic to the retrospective nature of this study and how our patient group was selected. Additionally, it is known that the risk of preterm childbirth is increased in mothers with personal history of asthma.^15^ Therefore, we may expect this specific patient population to have slightly higher incidence of a positive family history of asthma compared to the general population. Conversely, unlike previous studies, our study did not find maternal tobacco use during pregnancy to be significantly associated with recurrent wheezing. Tobacco exposure after birth, however, was nearly statistically significant in this study, which would be consistent with previous studies.^3,5,9^ These findings are likely due to our study being underpowered to ultimately detect this difference statistically.

The association specifically between perinatal exposures and the development of recurrent wheezing in preterm infants is less well established. We reviewed factors relating specifically to the newborn period and found few that associated with the development of recurrent wheezing in our study population. However, exposure to supplemental oxygen was significantly associated with the development of recurrent wheezing during the first 3 years of life in this study. Other studies have investigated this association previously in preterm infants without BPD, with conflicting results.^13,16^ This may be the result of differences in study design and patient populations and warrants additional investigation. The duration of supplemental oxygen exposure (0–48 vs. 48–96 vs. 96+ h) was not associated with higher odds of recurrent wheezing in our study, suggesting that exposure to oxygen even briefly may increase odds of developing bronchial hyperactivity, resulting in recurrent wheezing early in life. How this happens is not known, as preterm infants are exposed to several factors and rapidly changing environments throughout the early neonatal period in addition to having an immature respiratory system. However, episodes of hypoxia or respiratory distress requiring supplemental oxygen, and the subsequent exposure to it, may confer risk of wheezing in the first few years of life. Recent studies in newborn mouse and primary human airway smooth muscle cells suggest intermittent hyperoxia and hypoxia has a long-term detrimental effect on airway function that may involve oxidative stress.^17,18^ Again, this remains an area of interest for future prospective studies to better define the role of oxidative stress, coupled with a potentially immature respiratory system in this patient population.

While our study found that exposure to CPAP only for a brief duration (<24 h) in the perinatal period was significant statistically for developing recurrent wheezing in the first 3 years of life, CPAP requirement alone or CPAP of more prolonged duration was not associated with increased odds of developing recurrent wheezing. Furthermore, our multivariable logistic regression model (Table 3) did not find that brief CPAP requirement was associated with the development of wheezing in this study. However, we did find that the composite variable of exposure to any type of respiratory support—be it invasive mechanical ventilation, noninvasive ventilation, or oxygen, either alone or in combination—was a significant factor associated with developing recurrent wheezing. This again may be a result of effects on an immature respiratory system. However, as alveolar development continues through the first decade of life, early intervention with invasive ventilation, noninvasive positive pressure ventilation, or oxygen could reasonably alter this process and possibly confer a risk for developing future respiratory symptoms.^19,20^

Similarly, exposure to antibiotics during the perinatal period was a factor that resulted in an increased likelihood for recurrent wheezing in our study. The exposure to antibiotics during the first year of life has been reported to increase risk of pediatric asthma previously in nonpreterm infants.^21,22^ Whether preterm birth with subsequent antibiotic exposure in the immediate perinatal period increases this risk further is unknown. However, the fact that this exposure remained significant after adjustment for other confounding variables suggests a more direct effect. The process by which antibiotic exposure affects immune phenotype, Th1 and pro-inflammatory cytokine response, Th2 cell response, and host microbiome is understandably highly complex and largely unknown.^21,23,24^ It should also be noted that the clinical indication for receiving antibiotics in the perinatal period was not captured as part of this study. This will therefore continue to be an area of interest moving forward, particularly as it relates to long-term outcomes in preterm infants.

Of infants who presented with recurrent wheezing in our study, many presented initially in the setting of a viral illness to either the emergency department, urgent care, or primary care provider’s office, and had positive viral testing. A limitation of our study is that we were not able to capture the severity of the viral illness from our retrospective data. RSV was the most common virus identified, being found in over 50% of cases. Many studies have shown an association between early exposure to RSV and development of recurrent wheezing and asthma.^24–26^ Furthermore, previous studies have shown that late preterm infants who received early RSV prophylaxis had a 66% reduction in rates of recurrent wheezing by 3 years of age, compared with a nontreated and similarly matched age group.^7^ This implies that early RSV exposure can increase risk for asthma by early childhood. While our study did not include infants who received RSV prophylaxis, the association between early RSV infection and the development of chronic wheezing and asthma is again suggested here. Rhinovirus was the second most common pathogen at initial presentation in our patient population of recurrent wheezers. Like RSV, early infection with rhinovirus is known to increase risk for allergic diseases and asthma, and this was again suggested in our study.^24^

This study has several limitations, most of them inherent to the study design and methodology. The main limitation of this study is its retrospective nature. Our results rely on physician diagnosis or parental reports of wheezing, since this specific patient population is unable to perform pulmonary function testing to assess for the presence of true airway obstruction and bronchodilator responsiveness. Based on the definitions for recurrent wheezing in this study, gathering our study population also relied on adequate documentation of appropriate ICD-10 codes to capture preterm infants born during the designated 3-year period. Our study also is limited in that we were unable to capture any wheezing episodes that did not result in a clinical visit within our healthcare system. This is further reflected by the absence of data for any viral testing in patients who did not meet the criteria for developing recurrent wheezing in this study. Presumably, those who had symptomatic viral illnesses with wheezing would be more likely to seek medical attention, and this may partially explain why our study has a high proportion of patients who met the criteria for recurrent wheezing. This is a source of intrinsic bias in this study and a potential limitation to our findings. Furthermore, indications for certain interventions, including antibiotics, supplemental oxygen, or invasive or noninvasive mechanical ventilation, were not always entirely clear from chart review. Thus, we hope our study promotes future prospective studies to determine if these findings will predict recurrent wheeze in late preterm infants.

Since this retrospective data was collected from a single center, our findings may not necessarily generalize to other hospital settings or communities. This is reflected in the relatively large number of non-Hispanic White patients in our cohort, as compared to African American/Black and other races in this study. The reason for this discrepancy is not entirely clear. However, it could in part be due to our focus on late preterm infants in this study, therefore, excluding extremely premature infants. This is an important limitation to recognize, as previous studies have identified African American/Black race as a consistent risk factor for preterm birth in the United States.^11^ Furthermore, black and Hispanic children are at increased risk for development of asthma compared to white children.^12^ These groups are underrepresented in our study when compared to non-Hispanic White patients. Furthermore, our study did not discern what, if any, socioeconomic factors may be associated with an increased likelihood of a recurrent wheezing diagnosis. For example, the specific locations where wheezing encounters occurred (emergency department or urgent care versus primary care provider’s office) and insurance information was not documented to detect how the presenting chief complaint or differences in healthcare utilization may confound these results. Our study aimed more to investigate perinatal exposures in late preterm infants that may be associated with an increased likelihood of developing recurrent wheezing but are still important limitations to recognize. Further, as we focused on only late preterm infants, our findings should not be extrapolated to any other group of infants (early preterm or term), as we did not include these infants in our study.

In conclusion, our study suggests that exposure to respiratory support may be a risk factor for developing recurrent wheezing in late preterm infants. Additionally, early antibiotic exposure increased the odds of developing recurrent wheezing in this patient population. These data suggest that exposures in the immediate newborn period may impact the respiratory system and immune response, leading to increased likelihood of early life recurrent wheezing and possibly asthma development. As reported in previous studies, we found that family history of asthma is an independent, strong predictor for the development of recurrent wheezing. The association of perinatal antibiotic exposure and development of recurrent wheezing in our study is unique to preterm infants, as previous studies have not found this to be a risk factor in term infants.^13^ The other factors associated with recurrent wheezing identified in our study, however, are similar for late pre-term infants to those reported in term infants. Further prospective studies are needed to determine whether these factors independently lead to the development of early recurrent wheeze and subsequent asthma in late preterm infants.

## Figures and Tables

**TABLE 1:** Demographic and birth information for late preterm infants who did and did not develop recurrent wheezing.

	Recurrent wheezing (*n* = 210)	No recurrent wheezing (*n* = 104)	*p* Value
Sex, female, *n* (%)	53 (25%)	30 (29%)	.5
Race, *n* (%)			.11
Non-Hispanic White	131 (62%)	60 (58%)	
African American/Black	61 (29%)	40 (38%)	
Other (Hispanic, Asian)	18 (8.6%)	4 (3.8%)	
Gestational age			.8
32 weeks	28 (13%)	11 (11%)	
33 weeks	28 (13%)	14 (13%)	
34 weeks	42 (20%)	21 (20%)	
35 weeks	37 (18%)	15 (14%)	
36 weeks	75 (36%)	43 (41%)	
NICU LOS (days), median (IQR)	8 (1, 17)	7 (0, 17)	.1
Birth weight (g), mean (SD)	2378 (551)	2448 (626)	.3
C-section, *n* (%)	94 (45%)	49 (47%)	.7

Abbreviations: IQR, interquartile range; LOS, length of stay; SD, standard deviation.

**TABLE 2:** Clinical characteristics related to neonatal course between late preterm infants who did and did not develop recurrent wheezing.

	Recurrent wheezing (*n* = 210)	No recurrent wheezing (*n* = 104)	*p* Value
Positive family history of asthma, *n* (%)	109 (52%)	39 (38%)	**.014**
Positive maternal tobacco use, *n* (%)	30 (14%)	12 (12%)	.5
Received medications during neonatal course			
Antibiotics	53 (25%)	8 (7.7%)	**<.001**
Steroids	7 (3.3%)	0 (0%)	.10
Surfactant	15 (7.1%)	5 (4.8%)	.4
Albuterol	1 (0.5%)	1 (1.0%)	.6
Required Invasive Mechanical Ventilation	34 (16%)	10 (9.6%)	.11
Required CPAP	78 (37%)	29 (28%)	.11
Duration of CPAP (*n* = 107)			
<24 h	34 (16%)	7 (6.7%)	**.019**
≥24 h	44 (21%)	22 (21%)	>.9
Required supplemental oxygen	44 (21%)	11 (11%)	**.023**
Duration of supplemental oxygen (*n* = 54)			
0–48 h	19 (43%)	6 (55%)	.5
48–96h	8 (18%)	1 (9.1%)	.7
>96 h	16 (36%)	4 (36%)	>.9
Required any degree of respiratory support	98 (47%)	36 (35%)	**.042**
Tobacco exposure after birth	66 (31%)	22 (21%)	.056

*Note: p* < .05 are defined as statistically significant in this study, with bolded values noting a statistically significant difference between groups in noted exposure/history.

Abbreviation: CPAP, continuous positive airway pressure.

**TABLE 3:** Multivariable logistic regression model for development of recurrent wheezing.

Characteristic	OR	95% CI	*p* Value
Race			
Non-Hispanic White	-	-	
African American/Black	0.62	0.36, 1.06	.080
Other	2.60	0.87, 9.63	.11
NICU LOS (days)	0.99	0.98, 1.0	.081
Family history of asthma	1.70	1.01, 2.88	**.045**
Antibiotics received during NICU stay	3.90	1.70, 10.3	**.003**
CPAP < 24 h during NICU stay	2.19	0.94, 5.77	.086
Supplemental oxygen during newborn period	2.26	1.08, 5.05	**.036**
Smoke exposure after birth	1.73	0.97, 3.17	.067

*Note: p* < .05 are defined as statistically significant in this study, with bolded values noting a statistically significant difference between groups in noted exposure/history.

Abbreviations: CI, confidence interval; CPAP, continuous positive airway pressure; LOS, length of stay; NICU, neonatal intensive care unit; OR, odds ratio.

## Data Availability

Data sharing is not applicable to this article as no new data were created or analyzed in this study.
